# Novel DNA methylome biomarkers associated with adalimumab response in rheumatoid arthritis patients

**DOI:** 10.3389/fimmu.2023.1303231

**Published:** 2023-12-22

**Authors:** Ishtu Hageman, Femke Mol, Sadaf Atiqi, Vincent Joustra, Hilal Sengul, Peter Henneman, Ingrid Visman, Theodorus Hakvoort, Mike Nurmohamed, Gertjan Wolbink, Evgeni Levin, Andrew Y.F. Li Yim, Geert D’Haens, Wouter J. de Jonge

**Affiliations:** ^1^ Department of Gastroenterology and Hepatology, Amsterdam University Medical Centers (UMC), University of Amsterdam, Amsterdam, Netherlands; ^2^ Tytgat Institute for Liver and Intestinal Research, Amsterdam University Medical Centers (UMC), University of Amsterdam, Amsterdam, Netherlands; ^3^ Department of Rheumatology, Amsterdam Rheumatology and Immunology Center, Vrije Universiteit (VU) University Medical Center, Amsterdam, Netherlands; ^4^ Genome Diagnostics Laboratory, Department of Human Genetics, Amsterdam University Medical Centers (UMC), University of Amsterdam, Amsterdam, Netherlands; ^5^ Department of Vascular Medicine, Amsterdam University Medical Centers, University of Amsterdam, Amsterdam, Netherlands; ^6^ Horaizon BV, Delft, Netherlands; ^7^ Department of Surgery, University of Bonn, Bonn, Germany

**Keywords:** DNA methylation, rheumatoid arthritis, adalimumab, machine learning, therapy response

## Abstract

**Background and aims:**

Rheumatoid arthritis (RA) patients are currently treated with biological agents mostly aimed at cytokine blockade, such as tumor necrosis factor-alpha (TNFα). Currently, there are no biomarkers to predict therapy response to these agents. Here, we aimed to predict response to adalimumab (ADA) treatment in RA patients using DNA methylation in peripheral blood (PBL).

**Methods:**

DNA methylation profiling on whole peripheral blood from 92 RA patients before the start of ADA treatment was determined using Illumina HumanMethylationEPIC BeadChip array. After 6 months, treatment response was assessed according to the European Alliance of Associations for Rheumatology (EULAR) criteria for disease activity. Patients were classified as responders (Disease Activity Score in 28 Joints (DAS28) < 3.2 or decrease of 1.2 points) or as non-responders (DAS28 > 5.1 or decrease of less than 0.6 points). Machine learning models were built through stability-selected gradient boosting to predict response prior to ADA treatment with predictor DNA methylation markers.

**Results:**

Of the 94 RA patients, we classified 49 and 43 patients as responders and non-responders, respectively. We were capable of differentiating responders from non-responders with a high performance (area under the curve (AUC) 0.76) using a panel of 27 CpGs. These classifier CpGs are annotated to genes involved in immunological and pathophysiological pathways related to RA such as T-cell signaling, B-cell pathology, and angiogenesis.

**Conclusion:**

Our findings indicate that the DNA methylome of PBL provides discriminative capabilities in discerning responders and non-responders to ADA treatment and may therefore serve as a tool for therapy prediction.

## 1 Introduction

Rheumatoid arthritis (RA) is an autoimmune disorder characterized primarily by pain and inflammation of the joints. The etiology of RA is not fully elucidated; however, it is known to occur in genetically predisposed patients and is triggered by environmental factors, such as smoking (1). Current treatments for RA are divided into conventional disease-modifying anti-rheumatic drugs (csDMARDs), such as methotrexate, or biological DMARDs (bDMARDs), such as tumor necrosis factor-alpha (TNFα) inhibitors (infliximab, adalimumab, or etanercept), costimulation modifiers (abatacept), interleukin-6 inhibitors (tocilizumab), and B cell-depleting drugs (rituximab) (2) or target synthetic DMARDs (tsDMARDs), such as Janus kinase (JAK) inhibitors (3). Adalimumab (ADA) is a human recombinant IgG1 monoclonal antibody that binds to soluble and membrane-bound TNFα and is utilized as therapy for RA treatment and other immune-mediated diseases (IMIDs) such as axial spondyloarthritis and inflammatory bowel disease (4).

The efficacy and safety of ADA in RA patients have been established by multiple clinical trials and usage in the clinical practice, with approximately 60%–70% of ADA-treated patients exhibiting response as indicated by the Disease Activity Score in 28 Joints (DAS28) using erythrocyte sedimentation rate (ESR) at weeks 12 and 24 and/or presence of radiological progression (5, 6). Although ADA, among other TNF inhibitors, has improved the treatment of RA, patients discontinue ADA due to a lack of response or the development of adverse events (4, 7).

Common practice involves shifting to an alternative treatment regimen by initiating treatment with a different biological upon inadequate therapy response (3). Expectedly, this procedure is very inefficient and debilitating, as it can result in the patient’s progression toward uncontrolled disease, ultimately causing irreversible joint damage (5). Hence, there is an unmet need to predict response to treatment, as no clinically validated biomarkers currently exist (8). So far, several studies have sought to identify prognostic biomarkers in RA patients that predict treatment outcomes by interrogating the genetic polymorphisms (3, 9–12), microRNAs (13), and basic TNFα levels (3, 12). An increasing body of evidence suggests that epigenetic alterations, such as aberrant DNA methylation, are involved in the pathogenesis of inflammatory conditions such as in RA (14–17). DNA methylation occurs when a methyl group binds to a cytosine–phosphate–guanosine (CpG) dinucleotide. DNA methylation is a molecular mechanism that can affect gene transcription, especially seen in hypo- or hypermethylation of gene promotors, of which the majority reside within CpG-rich regions called CpG islands (18, 19). Complex immune-mediated diseases such as RA are thought to manifest in a genetically susceptible host, which manifests into dysregulated inflammatory processes in combination with the environment through epigenetic mechanisms (20–22). Furthermore, differentially methylated patterns have been reported in peripheral mononuclear blood cells, fibroblast-like synoviocytes, and synovial T cells of RA patients (15, 20, 23). DNA methylation in peripheral blood or methylome has therefore been proposed as a biomarker tool to predict therapy response in RA patients (24). Different studies investigated the association of the DNA methylome with response to methotrexate in RA patients (25–31). DNA methylation signatures in peripheral blood associated with response to anti-TNFα therapy (ADA, etanercept, infliximab, and golimumab) have been reported by Plant et al. (32), Julia et al. (33), and Tao et al. (34).

Here, we performed an exploratory epigenome-wide association study (EWAS) on whole peripheral blood (PBL) of RA patients who were scheduled to start ADA treatment where we explored whether a response to ADA could be predicted *a priori*. Through stability selection gradient boosting (35), we identified a 27-CpG classification model that was capable of predicting response before starting treatment.

## 2 Methods

### 2.1 Study design and response assessment

A retrospective cohort was assembled consisting of adult RA patients followed up between 2004 and 2018 who were scheduled to start ADA treatment at Reade, Expertise Center for Rehabilitation and Rheumatology, in Amsterdam, the Netherlands. Whole PBL was collected before the start of treatment whereupon patients were followed up as part of routine clinical care. A second visit was scheduled 3 to 6 months into treatment where therapy response was assessed based on the DAS28 score. The DAS28 is a clinical scoring tool on a 1–10-point scale that scores the swollen and/or painful joints together with ESR or C-reactive protein (CRP) levels and visual analog scale (VAS) disease activity of the patient, allowing for both clinical and biochemical response to be measured (36). A decrease of at least 1.2 points and/or a disease activity of less than 3.2 points was defined as response to therapy (**Table 1**). The assembly of this cohort was approved by the local Medical Ethics Committee of Slotervaart Hospital and Reade (NTR6868), and written informed consent was obtained from all patients prior to sampling.

**Table 1:** Therapy response criteria.

	Description
**Responders**	Therapy responders with a DAS28 < 3.2 (or decrease of at least 1.2 points)
**Non-responders**	Therapy non-responders with DAS28 > 5.1 (or decrease of <0.6 points)

Response was based on the DAS28 score (a 1–10-point scale) after a 3–6-month assessment (i.e., a decrease of at least 1.2 and/or reaching a disease activity of lower than 3.2). This is a clinical tool scoring the swollen and/or painful joints together with ESR or CRP levels and VAS disease activity of the patient. With this tool, the clinical and biochemical response to a biological agent can be measured.

DAS28, Disease Activity Score in 28 Joints; ESR, erythrocyte sedimentation rate; CRP, C-reactive protein; VAS, visual analog scale.

### 2.2 Sample collection, DNA isolation, and whole-genome DNA methylation profiling

PBL samples were collected prior to the start of ADA treatment in 6.0 mL BD EDTA vacutainer tubes and stored at −80°C. PBL samples were thawed, and genomic DNA (gDNA) was extracted using the QIAsympony (Qiagen, Valencia, CA, USA) at the Department of Human Genetics, Amsterdam UMC, according to manufacturer protocol. The FLUOstar OMEGA was used for assessing the quantity of the DNA. The gDNA (750 ng) was then randomly distributed across the plate to limit potential batch effects, after which gDNA was subjected to bisulfite conversion using the Zymo EZ DNA Methylation™ kit according to the manufacturer’s protocol, and the DNA was hybridized onto the Illumina HumanMethylationEPIC BeadChip array for whole-genome DNA methylation profiling (37).

### 2.3 DNA methylation data analysis

Data were analyzed following the pipeline previously published by de Krijger et al. (38). In brief, raw DNA methylation data were imported into R (version 4.2.0) using the Bioconductor package minfi (version 1.44) (39), followed by functional normalization (40) and quality control using the shinyMethyl package (version 1.34) (41). Probes that hybridized to allosomes were excluded from the analysis. Gaphunter was utilized to identify potential genetic variants by harnessing the bi- or triclustered pattern often presented by genetic variants by setting the threshold to 0.3 (42). M-values were used for statistical analysis and percentage methylation for visualization (43). Subsequent differential methylation analyses were performed through generalized linear regression analysis using the limma package (version 3.54) (44) where age, sex, concomitant methotrexate use, and smoking were adjusted to investigate whether these confounders affected the prediction algorithm. The ChAMP package (version 2.28) (45) was subsequently used for gene set enrichment analysis (GSEA). Visualizations were put together in ggplot (version 3.4) (46). CpGs of interest were annotated to genes according to the Illumina platform as well as based on their presence within a range of 20.000 from the nearest gene. For the hypothesis-driven approach, we sought to understand whether RA-associated differentially methylated genes also displayed ADA response-associated differences. To this end, we identified all CpGs annotated to the RA-associated genes— *CXCL12*, *DLGAP2*, *IL6*, *IL10*, *PRSS16*, and *STAT3*— which represent genes that were found to be RA-associated at the level of DNA methylation in a review by Ciechomska et al. (14). A summary p-value was calculated per gene by aggregating the p-values using the Brown method (47), a method often used in meta-analyses. Visualizations were generated using ggplot (version 3.4) (46) and ggbio (version 1.46.0) (48).

### 2.4 Blood cell estimation

The blood cell distribution was estimated from the DNA methylation data using the estimateCellCounts2 function from FlowSorted.Blood.EPIC (version 1.12.1) package (49) against the IDOL dataset (49), which contains DNA methylation profiles from B, CD4T, CD8T, monocytes, neutrophils, and NK cells. A quadratic programming approach was employed to predict the cellular composition per sample, and a two-way ANOVA test was conducted to statistically compare differences between groups.

### 2.5 Stability-selected gradient boosting analysis

To identify DNA methylation markers that classify therapy responders from non-responders before the start of treatment, extreme gradient boosting analysis with feature selection was used (38). This methodology was reported by de Krijger et al. (38). The data were split into a 70% training set and a 30% testing set. The classifier was trained through repeated cross-validation on the training set, where the performance was evaluated on the withheld test set. For optimization purposes, the area under the receiver operating characteristic (AUROC) scores were calculated for each repetition of the cross-validation and averaged for the final test AUROC. To select the most predictive CpGs, during each training fold, a random noise variable was introduced into the model. All features whose calculated feature importance exceeded the random variable were retained, whereas the features that scored less than the random variable were discarded. The resultant trees (n = 100), each containing its own set of ranked CpG markers according to relative importance, were then combined using pairwise permutation analysis (35).

### 2.6 Statistical analysis of clinical variables

Baseline characteristics of all included patients were summarized using descriptive statistics (**Table 2**). Categorical variables are presented as percentages and continuous variables as mean or median annotated with the standard deviation (SD) or interquartile range (IQR), respectively. Differences in distribution between responders, non-responders, and the different cohorts were assessed using a chi-square test (categorical variables), independent samples t-test, or the Mann–Whitney U (continuous variables). Two-tailed probabilities were used with a p-value of ≤0.05 considered statistically significant. Analyses of clinical data were performed in IBM SPSS statistics (version 26).

**Table 2:** Baseline characteristics of the patients.

	Responders (n = 49)	%	Non-responders (n = 43)	%	p-Value
Demographics					
**Age, *mean (SD)***	52.7	(9.3)	55.4	(12.5)	0.24
**Female, *n (%)***	39	(79.6)	32	(74.4)	0.56
**BMI, *mean (SD)***	25.3	(5.1)	25.9	(4.9)	0.62
**Smoking, *n (%)***	10	(20.4)	18	(42.9)	0.02
**Disease duration years, *median IQR***	10.6	(3.4–22.5)	6.7	(2.1–16.7)	0.20
**IgM-RF positive, *n (%)***	34	(70.8)	33	(76.7)	0.52
**ACPA positive, *n (%)***	33	(71.7)	27	(69.2)	0.80
**Erosive, *n (%)***	27	(57.4)	29	(67.4)	0.33
**DAS28 SJC at baseline, *median IQR***	6	(3.0–9.0)	5	(2.0–8.0)	0.15
**DAS28 TJC at baseline, *median IQR***	5	(3.0–10.5)	6.0	(2.0–11.0)	0.93
**CRP at baseline, *median IQR***	9	(3.0–32.0)	14	(5.8–27.8)	0.22
**ESR at baseline, *median IQR***	18	(9.0–36.5)	24.5	(14.3–39.0)	0.23
**Patient global assessment at baseline, *mean (SD)***	55.9	(21.2)	59.5	(21.2)	0.42
**DAS28 at baseline, *mean (SD)***	4.8	(1.2)	4.9	(1.3)	0.64
**DAS28 at wk 16, *mean (SD)***	2.6	(1.2)	3.8	(1.4)	<0.001
**Concomitant MTX, *n (%)***	42	(85.7)	28	(65.1)	0.02
**Concomitant prednisolone, *n (%)***	12	(24.5)	17	(39.5)	0.12
**TNFi naïve, *n (%)***	15	(31.3)	10	(25.0)	0.52

BMI, body mass index; ACPA, anti-citrullinated protein antibody; CRP, C-reactive protein; ESR, erythrocyte sedimentation rate; IgM-RF, IgM rheumatoid factor; DAS28, Disease Activity Score in 28 Joints; wk, week; MTX, methotrexate; TNFi, tumor necrosis factor-alpha inhibitor; SD, standard deviation; IQR, interquartile range; SJC, swollen joint count; TJC, tender joint count.

## 3 Results

### 3.1 Study population

The demographic and disease characteristics of patients are summarized in **Table 2**.** **A total of 92 RA patients were retrospectively included, from which whole PBL was stored before the start of ADA treatment. Patients were categorized as therapy responders (R) or non-responders (NR) based on the criteria described in **Table 1**, which yielded 49 and 43 responders and non-responders, respectively. At baseline, clinical (age (p = 0.24), sex (p = 0.56), and body mass index (BMI) (p = 0.62)), disease (IgM-RF positive (p = 0.80), erosive phenotype (p = 0.33), or disease activity-related (CRP (p = 0.22) and DAS28 (p = 0.64)) parameters differed significantly between responders and non-responders. By contrast, non-responders had significantly more smokers (p = 0.02) and, expectedly, higher DAS28 at week 16 (p < 0.001). Furthermore, a higher percentage of the responders had concomitant methotrexate (MTX) use in addition to ADA (p = 0.002).

### 3.2 Exploratory data analyses

**Figure 1:**
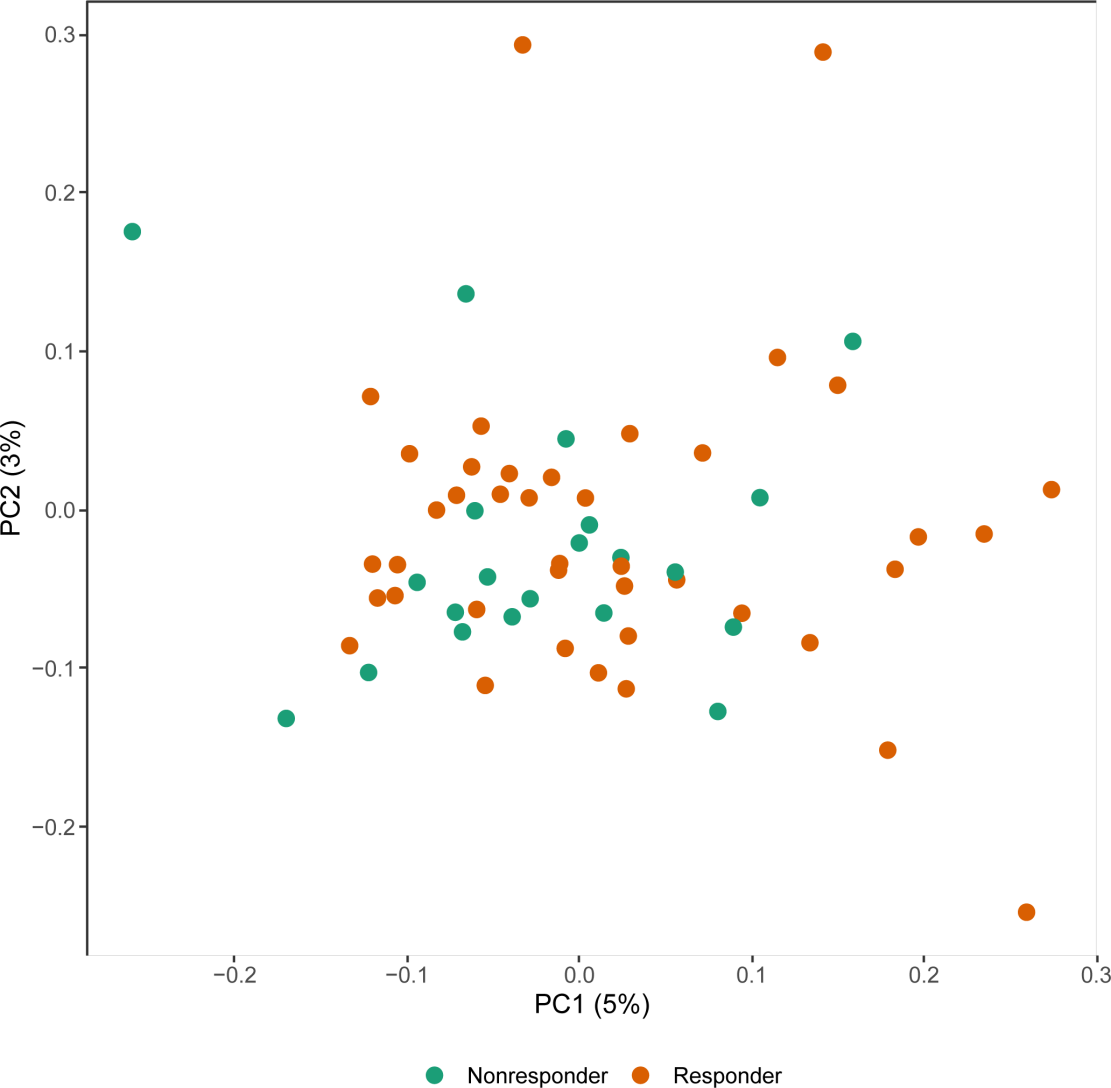
Principal component (PC) analysis of the methylome of rheumatoid arthritis (RA) therapy responders (orange) versus non-responders (green).

**Figure 2:**
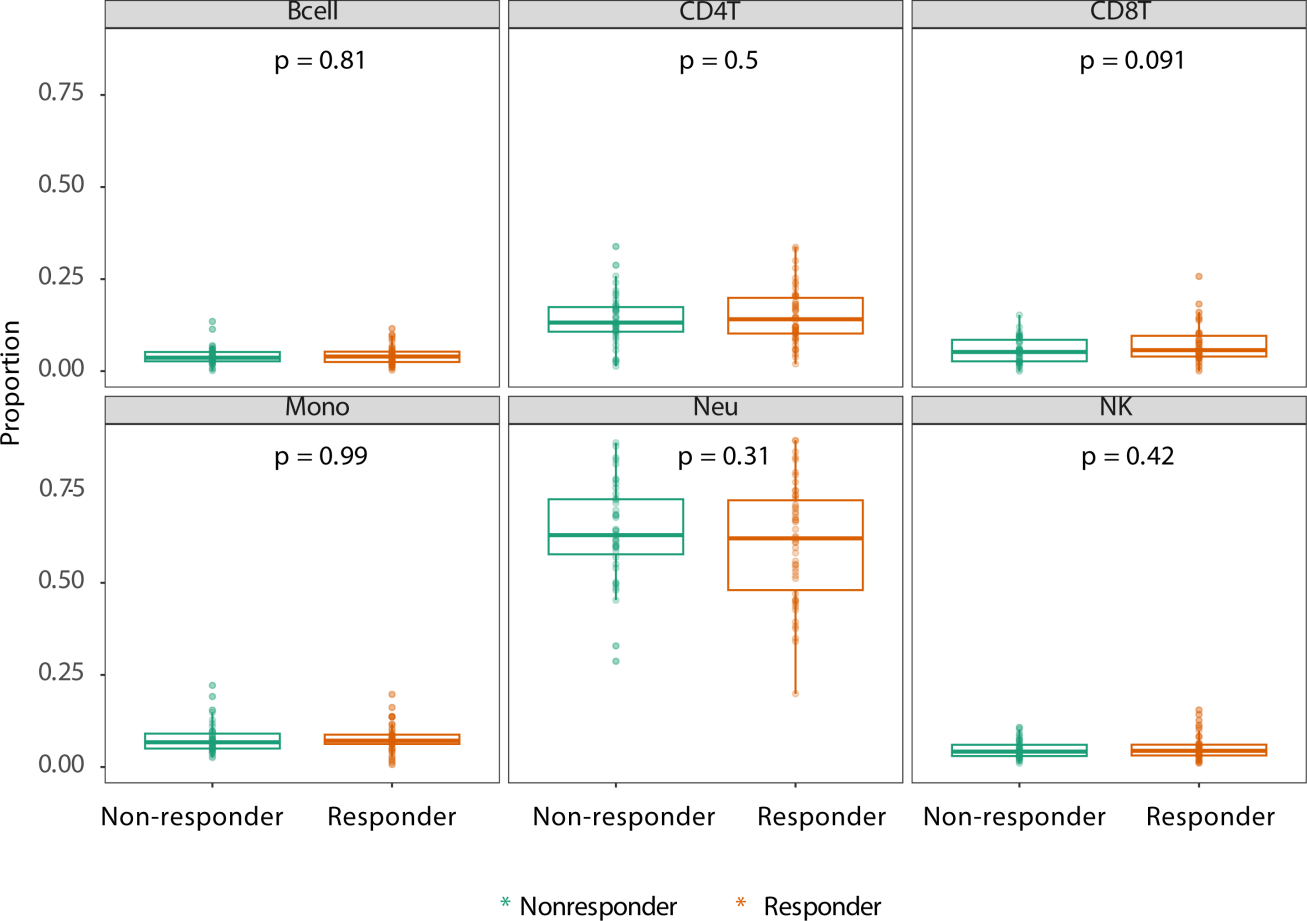
Estimated cell proportions as derived from Houseman algorithm cell mixture deconvolution from DNA methylation data of rheumatoid arthritis (RA) patients on adalimumab (ADA) treatment who are therapy responders (orange) and non-responders (green). The x-axis of each box illustrates the difference between RA responders and non-responders. p-Values are calculated using ANOVA testing. The y-axis demonstrates the proportion of reported cell type.

We first explored whether response to treatment was visible at an epigenome-wide level. Principal component (PC) analysis of the DNA methylome indicated no global differences between R and NR (**Figure 1**). Since we observed previously that significant differences existed in concomitant MTX use and smoking behavior at the time of sampling (**Table 2**), we assessed both by principal component analysis (PCA). Again, we detected no clustering according to the aforementioned confounders (**Supplementary Figure 1**). Since peripheral blood is composed of multiple different cell types, each of which has its own DNA methylation profile (50), we estimated the various cellular proportions and investigated whether differences were observable between responders and non-responders (**Figure 2**). There were no significant differences in the estimated cellular composition between R and NR with regard to the estimated B, CD4 T, CD8 T, monocytes, neutrophils, and NK populations.

### 3.3 Stability-selected gradient boosting predicts objective response to adalimumab

**Figure 3:**
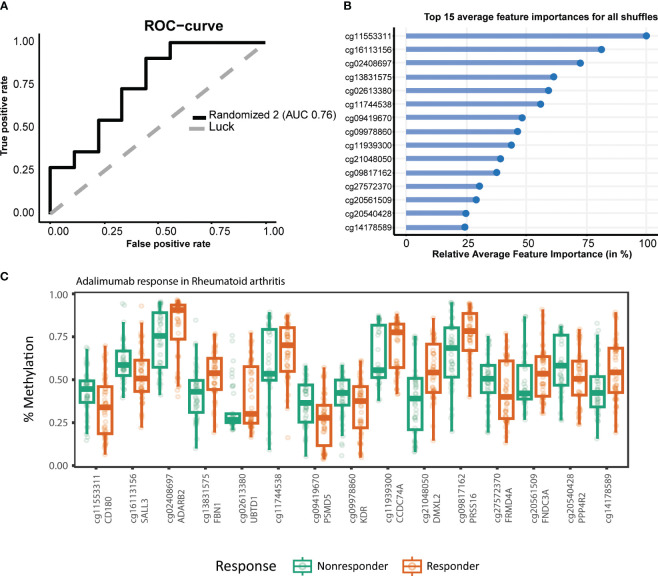
Stability-selected gradient boosting analysis performance of rheumatoid arthritis (RA) adalimumab (ADA) response prediction. **(A)** Receiver operating characteristic curve demonstrating an accuracy of 0.76 on the test set. The top 15 predictor CpGs visualized using **(B)** feature importance and **(C)** % methylation grouped by response.

**Figure 4:**
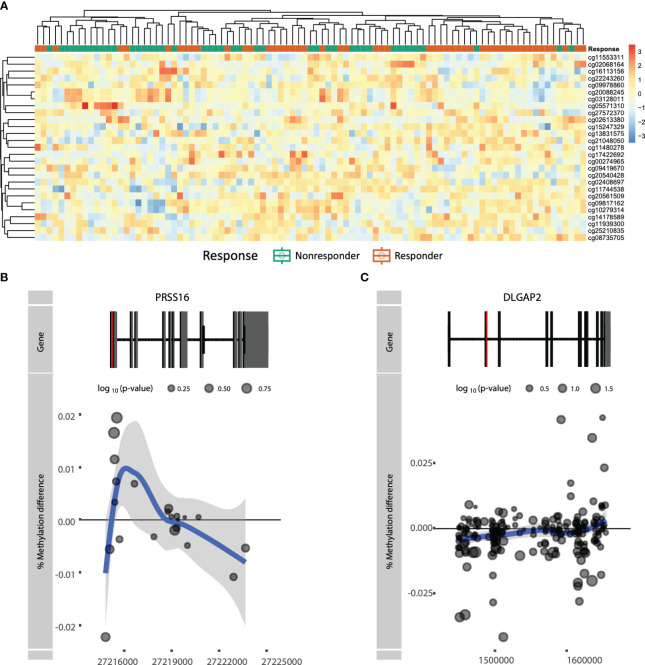
**(A)** Heatmap of actual methylation of the nominally significant predictor CpGs. Columns are sorted by hierarchical clustering and are colored by the response group: responders (orange) and non-responders (green). Visualization of genes **(B)** *PRSS16* and **(C)** *DLGAP2* by plotting the difference in mean % methylation on the y-axis relative to the position on the chromosome and the gene (“Gene”) on the x-axis. Dots represent probes on the Illumina HumanMethylationEPIC BeadChip array. The blue trend line represents the loess-smoothed average across all methylation probes for the indicated region with surrounding gray area representing the standard error. The first exon of the gene is represented in red.

To establish a prognostic predictive model of ADA response, we split the data into a 70% training and a 30% test set. We conducted stability-selected gradient boosting on the training data to define a prediction model that we subsequently validated against the test set. We observed that our best-performing classification model was capable of predicting prognostic response to therapy as evidenced by an AUROC of 0.76 (**Figure 3A**, **Table 3**). This classification model was composed of 27 CpGs (**Figures 3B, C**). Given the potential confounding by concomitant MTX use and smoking, we subsequently conducted a linear regression analysis on these 27 CpGs where we included concomitant MTX use, smoking, and sex and age as covariates (**Supplementary File 1**). Of the 27 CpGs, 20 presented p-values below 0.05, implying association with ADA response independent of concomitant MTX use and smoker behavior. Focusing on all 27 predictor CpGs, we found that hierarchical clustering of all samples did not show response-associated clustering, suggesting that the predictor probes were non-linearly associated with response (**Figure 4A**). Annotating all 27 CpGs, we found that 23 annotated to genes. Further interrogation of the 23 gene-bound CpGs indicated that response-associated hypermethylation was observed in the predictor CpGs annotated to genes *ADAP1*, *MRPL28*, *GNA12*, *UBTD1*, *OLIG2*, *CCDC74A*, *RPH3AL*, *PRSS16*, *MIR3143*, *H2BC12*, *DMXL2*, *FBN1*, and *ADARB2.* By contrast, response-associated hypomethylation was observed for *TARS*, *GSTM5*, *KIF19*, *PPP4R2*, *PSMD5*, *FRMDA4A*, *KDR*, *CD180*, *MAST4*, and *SALL3.* Interestingly, we identified multiple predictor CpGs within genes *PRSS16* (**Figure 4B**) and *DLGAP2* (**Figure 4C**). Overall, we observed that *PRSS16* showed response-associated hypermethylation in the transcription start site (TSS), which is where the two predictor CpGs (cg10279314 and cg09817162) were located. By contrast, *DLGAP2* demonstrated a more heterogeneous differential methylation pattern with hypomethylation near the TSS, which is where the two predictor CpGs (cg20088245 and cg03128011) were found, whereas the region of the gene downstream of the first exon (gene body) demonstrated a more dispersed differential methylated pattern. To understand the biological properties of our reported predictor CpGs, we performed GSEA to gain insight into the biological relevance of our reported predictor CpGs (**Supplementary Figure 2**). GSEA identified 83 significantly enriched processes (p < 0.05), with noteworthy hits related to immunological pathways such as “macrophage-enriched metabolic network” (1,075 genes), “immune (humoral) and inflammatory response” (504 genes), and “T-lymphocyte progenitors reprogrammed to natural killer cells” (276 genes) (**Supplementary Figure 2**, **Supplementary Table 1**).

**Table 3:** The 27 predictor CpG capable of distinguishing responders and non-responders.

CGID	chr	pos	Δ% Methylation	p-Value	Annotated gene	Gene feature	Direction R *vs.* NR
**cg02068164**	chr5	33439794	−0.059	1.01E−01	*TARS*	TSS	Hypomethylation
**cg20088245**	chr8	1321375	−0.087	2.77E−02	*DLGAP2*	TSS	Hypomethylation
**cg08735705**	chr7	1003645	0.038	2.54E−01	*ADAP1*	Enhancer	Hypermethylation
**cg22243260**	chr3	126946036	−0.069	2.93E−02	*NA*	NA	Hypomethylation
**cg10279314**	chr6	27185896	0.069	1.61E−02	*PRSS16*	Intron	Hypermethylation
**cg25210835**	chr1	110254828	−0.060	1.35E−01	*GSTM5*	Promoter	Hypomethylation
**cg17422692**	chr16	420245	0.079	2.23E−02	*MRPL28*	Promoter	Hypermethylation
**cg03128011**	chr8	1321333	−0.077	3.38E−02	*DLGAP2*	Promoter	Hypomethylation
**cg05571310**	chr17	72350354	−0.077	1.03E−02	*KIF19*	Intron	Hypomethylation
**cg20540428**	chr3	73045686	−0.064	4.53E−02	*PPP4R2*	Promoter	Hypomethylation
**cg15247329**	chr7	2764246	0.035	2.38E−01	*GNA12*, *AMZ1*	Intron	Hypermethylation
**cg02613380**	chr10	99330076	0.075	4.14E−02	*UBTD1*	Promoter	Hypermethylation
**cg09419670**	chr9	123605666	−0.097	9.50E−04	*PSMD5*	Intron	Hypomethylation
**cg20561509**	chr13	49427965	0.058	9.06E−02	*FNDC3A*	Promoter	Hypermethylation
**cg27572370**	chr10	14002394	−0.073	3.25E−02	*FRMD4A*	Intron	Hypomethylation
**cg09978860**	chr4	56023921	−0.053	7.98E−02	*KDR*	Intron	Hypomethylation
**cg00274965**	chr21	34405681	0.105	1.90E−02	*OLIG1*	Intron	Hypermethylation
**cg11939300**	chr2	132584904	0.066	5.58E−02	*CCDC74A*	Intron	Hypermethylation
**cg14178589**	chr6	168726836	0.102	5.94E−03	*NA*	NA	Hypermethylation
**cg11480278**	chr17	83580	0.108	4.56E−04	*RPH3AL*	Unknown	Hypermethylation
**cg09817162**	chr6	27185676	0.121	1.44E−03	*PRSS16*	Enhancer	Hypermethylation
**cg11553311**	chr5	66541588	−0.083	7.08E−03	*CD180*, *(MAST4)*	Unknown	Hypomethylation
**cg21048050**	chr15	51912957	0.156	7.52E−05	*DMXL2*	Promoter	Hypermethylation
**cg13831575**	chr15	48834416	0.105	9.33E−04	*FBN1*	Promoter	Hypermethylation
**cg11744538**	chr17	42646995	0.089	4.27E−02	*NA*	NA	Hypermethylation
**cg02408697**	chr10	1416920	0.089	6.05E−03	*ADARB2*	Intron	Hypermethylation
**cg16113156**	chr18	76266265	−0.075	2.01E−02	*SALL3*	Unknown	Hypomethylation

CGID, Illumina CpG ID; chr, c hromosome; pos, position on human genome (hg19); Δ% Methylation, d ifference in percentage methylation; p-value, p-value associated with difference percentage methylation; Annotated gene, g ene closest to the CpG, and NA was used if no gene was annotated based on Illumina’s metadata; Gene feature, g enetic feature encompassing the CpG, including TSS (transcription start site), enhancer, introns, exons, or unknown; Direction R vs. NR, t he direction of the effect relative to non-responders, and o rder was based on the feature importance.

### 3.4 Rheumatoid arthritis-associated differentially methylated genes also present response-associated differences in DNA methylation

**Figure 5:**
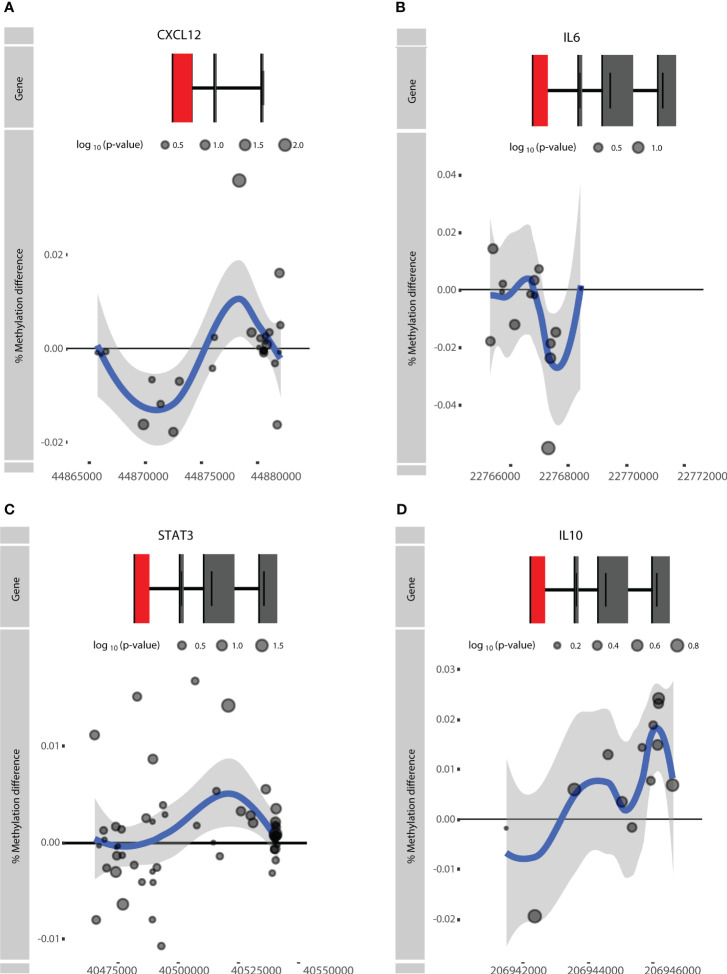
Visualization of the rheumatoid arthritis (RA)-associated genes **(A)** *CXCL12*, **(B)** *IL6*, **(C)** *STAT3*, and **(D)** *IL10.* Difference in % methylation is plotted on the y-axis relative to the position on the chromosome and the gene (“Gene”) on the x-axis. Dots represent probes on the Illumina HumanMethylationEPIC BeadChip array. The blue trend line represents the loess-smoothed average across all methylation probes for the indicated region with surrounding gray area representing the standard error. The first exon of the gene is represented in red.

We next investigated whether genes identified in previous RA DNA methylation studies displayed response-associated differential methylation as well. To this end, we exercised a hypothesis-driven approach where we examined the methylation status of previously reported genes described in *STAT3*, *CXCL12*, *IL10*, and *IL6*, as discussed in the review by Ciechomska et al. (14, 15). It was found that *STAT3*, *CXCL12*, and *IL6* were hypomethylated in RA patients relative to non-RA individuals and that *IL10* was hypermethylated (14). Interrogating these genes within the context of response to ADA revealed that *CXCL12* and *IL10* displayed hypomethylation in the TSS, whereas hypermethylation was seen within the gene body of both genes (**Figures 5A, D**). By contrast, *IL6* presented hypomethylation within the gene body and in the intragenic region, but hypermethylation near the TSS (**Figure 5B**). Notably, *STAT3* showed no distinct methylation pattern (**Figure 5C**).

## 4 Discussion

This study aimed to identify CpGs whose DNA methylation level was capable of predicting response to ADA therapy in RA patients prior to the start of treatment. Response to therapy was defined based on the DAS28 score assessed over a treatment period of 3 to 6 months (36). Through supervised stability-selected extreme gradient boosting, we were able to identify 27 CpGs whose DNA methylation collectively predicted response to therapy. Several of the predictor CpGs were annotated to genes that had previously been implicated in RA or general inflammatory processes, with a particular focus on T-cell biology. This corroborates observations made by Bek et al., where genetic variants associated with anti-TNFi response were found to map to genes involved in T-cell function (9). Multiple predictor CpGs were identified in *PRSS16* and *DLGAP2*. *PRSS16* is a gene whose protein is associated with gout, a form of inflammatory arthritis. *PRSS16* mutations have been characterized by monosodium urate deposition that leads to inflammasome and subsequent cytokine production (51). *PRSS16* maps to the extended HLA class I region (52). Moreover, *PRSS16* is highly expressed in the cortex of the thymus and is proposed to be involved with T-cell development in the thymus, in particular the positive selection of T cells (53). Notably, cortical thymic epithelial cells contribute to the positive selection of T cells via antigen presentation. Interestingly, when aberrant positive selection occurs, autoimmunity is found in mouse models (54). Taken together, *PRSS16* is proposed as a candidate gene for auto-inflammatory diseases. *DLGAP2* encodes a membrane-associated protein that has been implicated in neuronal cells (55). While *Dlgap2* has been implicated as being differentially methylated in both aging and osteoarthritis in mice (56), no further link with ADA or RA can be identified in the literature. Other genes that were found to harbor predictor CpGs included *TARS*, *KDR*/*VEGFR2*, and *CD180. TARS* encodes a threonyl-tRNA synthetase implicating a role in amino acid processing. Despite their household role, several CpGs within the genes were found to be differentially methylated between RA patients and non-RA individuals (17). *KDR/VEGFR2* encodes vascular endothelial growth factor receptor 2 (*VEGFR2*), a receptor to VEGF. VEGF is responsible for endothelial activation, endothelial growth, and angiogenesis (57), where angiogenesis is one of the key pathways for the synovial tissue expansion in RA and is accompanied by a sustained inflammatory process in the synovial tissue characterized by proinflammatory cytokines and upregulated levels of VEGF in synovial tissue (57, 58). Accordingly, targeting angiogenesis in RA has been proposed as a treatment strategy for RA (1). *CD180* encodes cluster of differentiation 180, a toll-like receptor homologue expressed mainly on B cells. Differential expression of CD180 was associated with other rheumatic diseases (59), such as systemic sclerosis (60), systemic lupus erythematosus (61), and Sjögren’s syndrome (62). While such a difference in either protein or gene expression of CD180 has not been reported for RA thus far, it stands to reason that CD180 might play a role in RA and its response to ADA.

The main strengths of our study lie in the strict patient selection criteria for response and non-response, which we based on the European Alliance of Associations for Rheumatology (EULAR) criteria. Furthermore, we explored the predictive features of the DNA methylome in a large RA patient cohort for ADA therapy response using extreme gradient boosting analysis, a state-of-the-art machine learning tool (35, 38). We intentionally investigated DNA methylation as a predictive biomarker for therapy response in peripheral blood since this material is easy to obtain in the context of developing an accessible diagnostic test. There are several limitations to address. First, concomitant MTX use may potentially exert an influence on the methylome as evidenced by prior investigations that demonstrated global DNA hypomethylation within blood cell populations, such as T cells and monocytes, isolated from RA patients following MTX treatment (25, 26, 63). Second, we were not able to perform gene expression on our own data set and could only theorize about the biology underlying the predictor CpGs. Third, to properly validate the performance of our predictive model, a properly setup validation cohort would need to be set up in an independent RA cohort. Several studies have reported differential methylated positions that distinguish therapy responders from non-responders treated with ADA, such as Tao et al. (34), where they performed a genome-wide epigenome association study on peripheral blood mononuclear cells (PBMCs) of RA patients treated with anti-TNFα medications such as ADA and etanercept. However, since we performed EWASs on whole blood patient materials, pooling our cohorts could lead to bias since the sample type is dissimilar. Fourth, since DNA methylation as an epigenetic mark is cell type-specific and peripheral blood is composed of different cell types, it is unclear whether the observed differential methylation signal is the result of actual DNA methylation or differences in cellular composition. While cellular composition can be largely estimated using the DNA methylome (50), such methods are often limited to the major cell populations. When interrogating these estimated cell proportions, we did not observe any response-associated differences in the estimated cellular composition. Finally, as DNA methylation measurements are conducted by “stamping” unmethylated cytosines into the genome through cytosine deamination, actual genetic variants can interfere with the methylation signal (64). However, we did not observe the characteristic tri- or bi-modal distribution of the methylation signal typically observed when interrogating genetic variants (42, 64).

Our results provide an initial, exploratory step toward the development of ADA response prediction in RA but require extensive validation in subsequent larger studies. We envision that future research can harness our data with the aim of developing a clinically applicable biomarker. Such a prognostic tool based on robust, validated, response-associated CpGs would reshape current clinical practice for RA, enabling treating clinicians to tailor medication to the patient and improving patient outcomes.

## Data availability statement

The datasets presented in this study can be found in online repositories. The names of the repository/repositories and accession number(s) can be found below: EGA EuropeanGenome-Phenome Archive, accession number EGAD00010002610 (https://ega-archive.org/studies/EGAS00001007578); https://zenodo.org, 10225486.

## Ethics statement

The studies involving humans were approved by Medical Ethics Committee of Slotervaart Hospital and Reade. The studies were conducted in accordance with the local legislation and institutional requirements. The participants provided their written informed consent to participate in this study.

## Author contributions

IH: Conceptualization, Formal analysis, Methodology, Writing – original draft, Writing – review & editing. FM: Conceptualization, Formal analysis, Methodology, Writing – original draft, Writing – review & editing. SA: Conceptualization, Formal analysis, Methodology, Writing – original draft, Writing – review & editing. VJ: Conceptualization, Formal analysis, Methodology, Writing – review & editing. HS: Writing – review & editing. PH: Writing – review & editing. IV: Writing – review & editing. TH: Writing – review & editing. MN: Writing – review & editing. GW: Writing – review & editing. EL: Writing – review & editing. AL: Conceptualization, Formal analysis, Methodology, Writing – original draft, Writing – review & editing. GD: Conceptualization, Writing – review & editing. Wd: Conceptualization, Writing – review & editing.

## Supplementary material

Supplementary Figure 1 Principal component analysis (PCA) of the methylome of RA therapy responders (orange) versus non-responders (green) and concomitant MTX users (triangle) or MTX naïve patients (circle).

Supplementary Figure 2 Gene set enrichment analysis (GSEA) against the gene ontology (GO) gene sets of our reported predictor CpGs (adjusted ρ<0.05).

Supplementary File 1 HTML Markdown output of the script used to perform the analysis.

Supplementary Table 1 Gene set enrichment analysis output of the predictor CpGs against GO gene sets. Columns represent the GO term, number of genes enriched in this pathway, area under the receiver operator curve of the Wilcoxon test, p value calculated using the Wilcoxon test, p value calculated using the Known Population Median Test, the Benjamini-Hochberg-adjusted p-value, and the pathway description. Table is ranked by p-value (low to high).
