# Supplementary figures and images for: Novel DNA methylome biomarkers associated with adalimumab response in rheumatoid arthritis patients
Source: Front Immunol. 2023 Dec 22;14:1303231. doi: 10.3389/fimmu.2023.1303231 (PMC10771853; doi:10.3389/fimmu.2023.1303231)

Active smoker

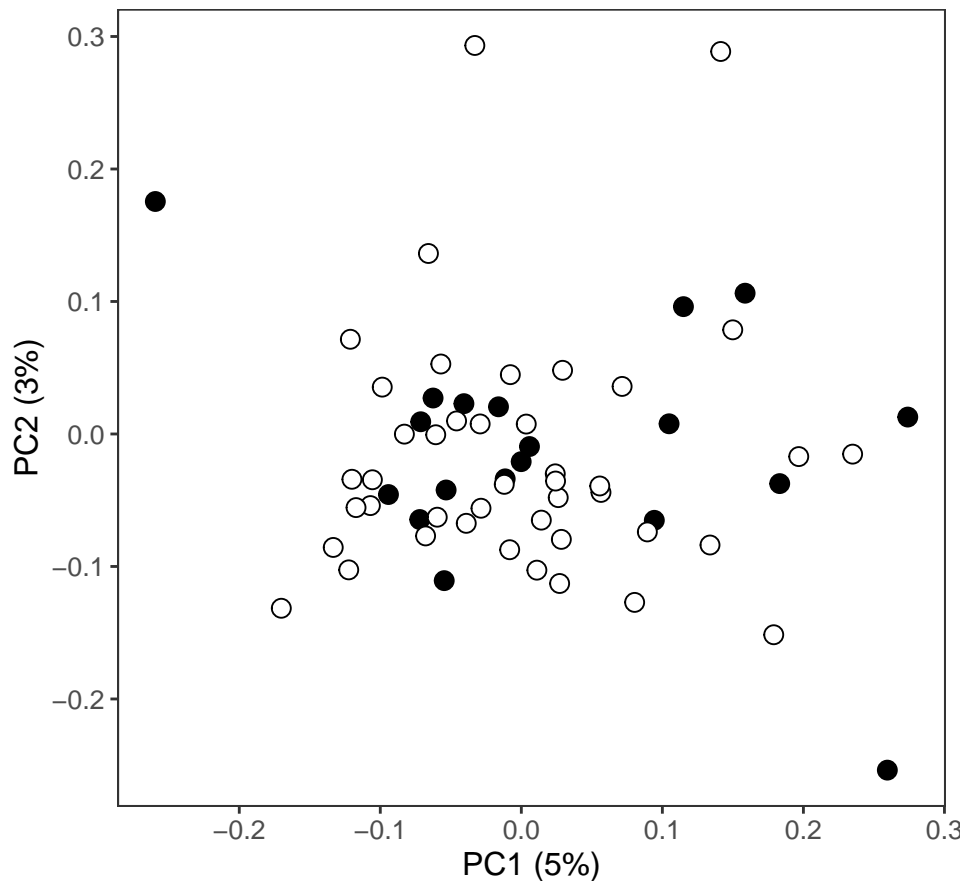

Concomitant methotrexate use

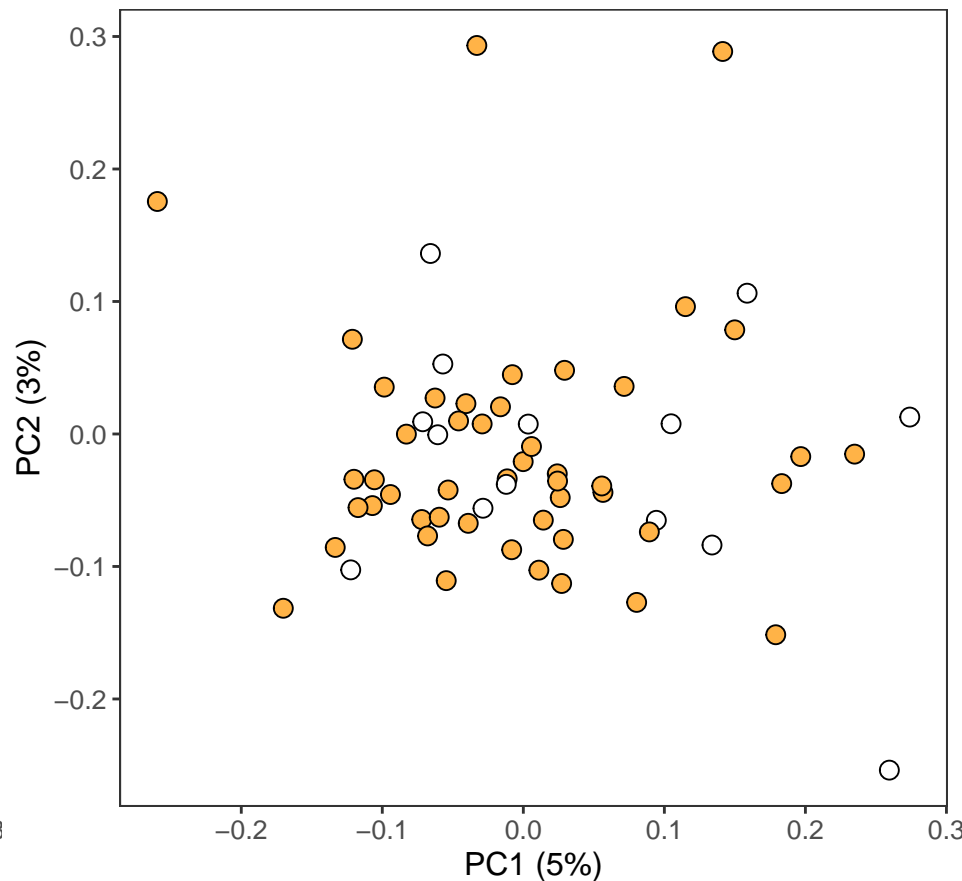

Supplement: Supplementary Figure 1 — Principal component analysis (PCA) of the methylome of RA therapy responders (orange) versus non-responders (green) and concomitant MTX users (triangle) or MTX naïve patients (circle). [file Image_1.pdf]

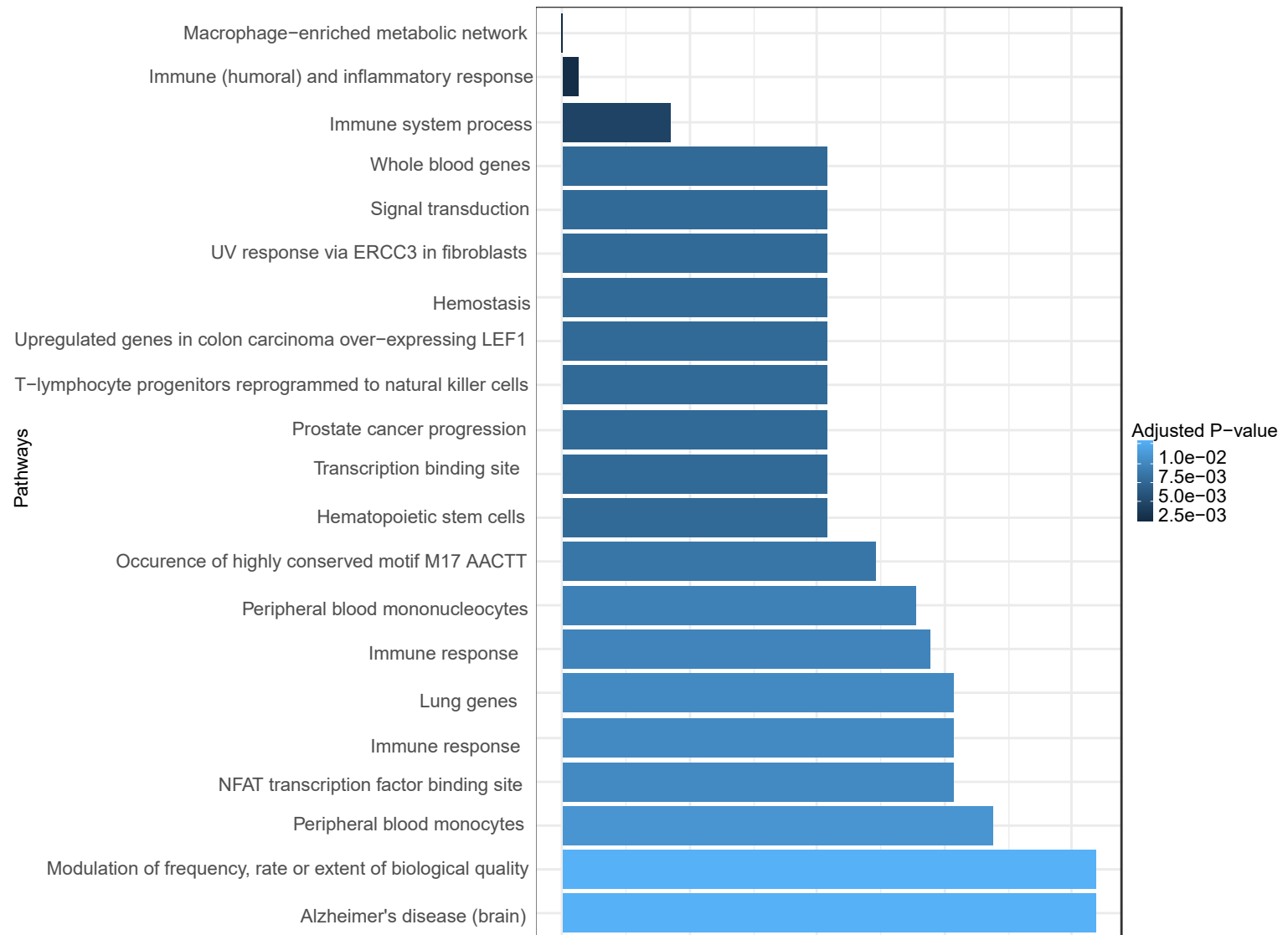

Supplement: Supplementary Figure 2 — Gene set enrichment analysis (GSEA) against the gene ontology (GO) gene sets of our reported predictor CpGs (adjusted ρ<0.05). [file Image_2.pdf]
