# Supplementary material for: Novel DNA methylome biomarkers associated with adalimumab response in rheumatoid arthritis patients
Source: Front Immunol. 2023 Dec 22;14:1303231. doi: 10.3389/fimmu.2023.1303231 (PMC10771853; doi:10.3389/fimmu.2023.1303231)
Supplement: Supplementary File 1 — HTML Markdown output of the script used to perform the analysis. [file DataSheet_1.pdf]

Novel DNA methylome biomarkers associate with adalimumab response in rheumatoid arthritis patients

Femke Mol

2023-09-22

In this Markdown file you will find the R script used for the analysis of the paper “Novel DNA methylome biomarkers associate with adalimumab response in rheumatoid arthritis patients”. This file only contains script, figures can be found in the manuscript. We analyzed Illumina EPIC BeadChip data of 93 rheumatoid arthritis (RA) patients, focussing on the differences in methylation between responders and nonresponders to adalimumab.

The following libraries were used:

```
library(preprocessCore)
library(minfi)
library(dplyr)
library(readxl)
library(IlluminaHumanMethylationEPICanno.ilm10b4.hg19)
library(missMethyl)
library(shinyMethyl)
library(ExperimentHub)
library(FlowSorted.Blood.EPIC)
library(ggplot2)
library(ggrepel)
library(ggrastr)
library(Cairo)
library(limma)
library(Gviz)
library(DMRcate)
library(ChIPpeakAnno)
library(org.Hs.eg.db)
library(GenomicFeatures)
library(TxDb.Hsapiens.UCSC.hg19.knownGene)
library(ChAMP)
```

Load in data and Quality control

Loading the data files with minfi.

```
## ancillary data
pmprobes <- read.csv("/mnt/smb/lpersonal/femke.mol/RA_ADA/data/Non-specific-probes-illuminaEPIC.csv")
## annotation data
anno_gr <- makeGRangesFromDataFrame(minfi::getAnnotation(IlluminaHumanMethylationEPICanno.ilm10b4.hg19),
    keep.extra.columns = T, seqnames.field = "chr", start.field = "pos", end.field = "pos")
## data samplesheet
dataDirectory <- "/mnt/smb/lpersonal/femke.mol/RA_ADA/data"
list.files(dataDirectory, recursive = TRUE)
meta_aumc <- read_xlsx("/mnt/smb/lpersonal/femke.mol/RA_ADA/data/20221027_samplesheet_aumc_FM.xlsx")
reade_mtx <- read_xlsx("/mnt/smb/lpersonal/femke.mol/RA_ADA/data/Humira_RA_finalselection_MTX.xlsx")
colnames(reade_mtx) <- c("Box_position", "Buffycoat_selection", "Sample_ID", "Prot",
    "Sample_date", "MTX")
meta_aumc$MTX <- reade_mtx$MTX[match(meta_aumc$Sample_ID, reade_mtx$Sample_ID)]
samplesheet_aumc <- minfi::read.metharray.sheet(dataDirectory, "20230818_samplesheet_aumc_FM.csv")

## amc data metharray (using samplesheet)
rgset_aumc <- minfi::read.metharray.exp(targets = samplesheet_aumc, recursive = T)

## predictor probes found by HorAIzon
horaizon_markers <- readxl::read_excel("/mnt/smb/lpersonal/femke.mol/RA_ADA/data/220704_Selected_markers_annotated.xlsx")
top15 <- c("cg11553311", "cg16113156", "cg02408697", "cg13831575", "cg02613380",
    "cg11744538", "cg09419670", "cg09978860", "cg11939300", "cg21048050", "cg09817162",
    "cg27572370", "cg20561509", "cg20540428", "cg14178589")
top15_xgb <- filter(horaizon_markers, CGID %in% c("cg11553311", "cg16113156", "cg02408697",
    "cg13831575", "cg02613380", "cg11744538", "cg09419670", "cg09978860", "cg11939300",
    "cg21048050", "cg09817162", "cg27572370", "cg20561509", "cg20540428", "cg14178589"))
top15_xgb <- top15_xgb[match(c("cg11553311", "cg16113156", "cg02408697", "cg13831575",
    "cg02613380", "cg11744538", "cg09419670", "cg09978860", "cg11939300", "cg21048050",
    "cg09817162", "cg27572370", "cg20561509", "cg20540428", "cg14178589"), top15_xgb$CGID),
    ]

secondary_split <- horaizon_markers %>%
    dplyr::filter(Cohort == "Secondary_split")
```

Quality contro (QC) was performed with shinyMethyl

```
rgset_aumc_ss <- shinyMethyl::shinySummarize(rgset_aumc)
shinyMethyl::runShinyMethyl(rgset_aumc_ss)

### There are some brain samples in the data, remove these
rgset_aumc_nobrain <- rgset_aumc[, pData(rgset_aumc)$gDNA_ID2D != "Brain"]
```

Blood cell distribution

After QC, we looked into the blood cell estimation using the method of Salas et al (2018). They used an updated version of the Houseman algorithm (2012), in which methylation data is used to estimate the blood cell distribution.

```
hub <- ExperimentHub()
FlowSorted.Blood.EPIC <- hub[["EH1136"]]

cellDist_aumc_salas <- estimateCellCounts2(rgset_aumc, cellTypes = c("CD8T", "CD4T",
    "NK", "Bcell", "Mono", "Neu"), compositeCellType = "Blood", processMethod = "preprocessNoob",
    probeSelect = "IDOL", referencePlatform = c("IlluminaHumanMethylationEPIC"))

cellDist_aumc_salas.df <- data.frame(cellDist_aumc_salas$prop, response = pData(rgset_aumc)$Response,
    array = rownames(cellDist_aumc_salas$prop))
# write.csv(cellDist_aumc_salas.df,
# '~/RA_ada/output/cellDist_aumc_salas.df.csv')

cellDist_aumc_salas_plot <- cellDist_aumc_salas.df %>%
    tidyr::pivot_longer(-c(array, response), names_to = "Celltype", values_to = "Proportion") %>%
    dplyr::filter(!is.na(response)) %>%
    ggplot(aes(x = response, y = Proportion, col = response)) + geom_boxplot(alpha = 0.25,
    show.legend = F) + geom_point(alpha = 0.25, show.legend = F) + facet_wrap(~Celltype) +
    xlab("Response") + theme_bw() + scale_colour_brewer(palette = "Dark2") + theme(legend.title = element_blank(),
    panel.grid.major = element_blank(), panel.grid.minor = element_blank(), legend.position = "bottom",
    text = element_text(size = 12))
print(cellDist_aumc_salas_plot)
```

No significant differences in cellular composition between responders and non-responders.

Normalization

Normalization of data is performed using preprocessFunnorm(). This is a between-array normalization method for Illumina platforms, which removes unwanted variation by regressing out variability explained by the control probes present on the array.

```
gmset_aumc <- preprocessFunnorm(rgSet = rgset_aumc)

gmset_aumc_nobrain <- preprocessFunnorm(rgSet = rgset_aumc_nobrain)
gmset_aumc_processed <- gmset_aumc_nobrain[-which(minfi::getAnnotation(gmset_aumc_nobrain)$chr %in%
    c("chrX", "chrY")), ]

gmset_aumc_filtered <- gmset_aumc_processed[!names(gmset_aumc_processed) %in% pmprobes,
    ]
gmset_aumc_filtered <- gmset_aumc_filtered[-which(getM(gmset_aumc_filtered) == -Inf,
    arr.ind = T)[, 1], ]

betas_aumc <- getBeta(gmset_aumc_filtered)
mvals_aumc <- getM(gmset_aumc_filtered)
```

We also check for gender mismatches:

```
table(pData(gmset_aumc_filtered)$Sex, pData(gmset_aumc_filtered)$predictedSex)

sexmismatch <- rbind(data.frame(pData(gmset_aumc_filtered)[which(pData(gmset_aumc_filtered)$Sex ==
    "Female" & pData(gmset_aumc_filtered)$predictedSex == "M"), ]), data.frame(pData(gmset_aumc_filtered)[which(pData(gmset_
aumc_filtered)$Sex ==
    "Male" & pData(gmset_aumc_filtered)$predictedSex == "F"), ]))

Cairo(width = 1500, height = 1000, type = "pdf", file = file.path(edaDir, "sexmismatch.pdf"),
    bg = "white", units = "px", dpi = 120)
data.frame(pData(gmset_aumc_filtered)) %>%
    dplyr::mutate(sexmismatch = ifelse(substring(Sex, first = 1, last = 1) != predictedSex,
        "Mismatch", "Match"), label = ifelse(sexmismatch == "Mismatch", Sample_ID,
        NA)) %>%
    ggplot(aes(x = xMed, y = yMed, col = Sex)) + geom_point() + geom_label_repel(aes(label = label),
    show.legend = F) + labs(title = "Predicted Sex", subtitle = "Median signal intensity entire chromosome",
    y = "ChrY", x = "ChrX", ) + theme_bw()
dev.off()
```

No mismatches detected.

Principal Component Analysis

We continue with the principal component analysis (PCA)

```
mvals_aumc_dm <- mvals_aumc - rowMeans(mvals_aumc)
mvals_aumc_dm_t <- t(mvals_aumc_dm)  #transpose to get cpg in column and sample in row (otherwise it will not work)
mvals_aumc_svd <- svd(mvals_aumc_dm_t)
mvals_aumc_svd_expvar <- round(mvals_aumc_svd$d/sum(mvals_aumc_svd$d), 2) * 100

mvals_aumc_svd_df <- data.frame(Sample_ID = pData(gmset_aumc_filtered)$gDNA_ID2D,
    Response = pData(gmset_aumc_filtered)$Response, Sex = pData(gmset_aumc_filtered)$predictedSex,
    Age = pData(gmset_aumc_filtered)$Age, MTX = pData(gmset_aumc_filtered)$MTX, Medication = pData(gmset_aumc_filtered)$Medi
cation,
    PC1 = mvals_aumc_svd$u[, 1], PC2 = mvals_aumc_svd$u[, 2])

ada_mvals_aumc_svd_df <- subset(mvals_aumc_svd_df, Medication == "Adalimumab")

mvals_aumc_svd_plotobj <- ggplot(mvals_aumc_svd_df, aes(x = PC1, y = PC2, Label = Sample_ID,
    Response = Response, Sex = Sex, Age = Age, Medication = Medication, MTX = MTX)) +
    geom_point(aes(col = Response, shape = factor(MTX)), size = 3) + theme_bw() +
    scale_colour_brewer(palette = "Dark2") + scale_shape_discrete(labels = c("No MTX",
    "MTX")) + xlab(paste0("PC1 (", mvals_aumc_svd_expvar[1], "%)")) + ylab(paste0("PC2 (",
    mvals_aumc_svd_expvar[2], "%)")) + theme(text = element_text(size = 12), legend.position = "bottom",
    panel.grid.major = element_blank(), panel.grid.minor = element_blank(), legend.title = element_blank())
# ggsave('/mnt/smb/lpersonal/femke.mol/RA_ADA/output/PCA_MTXandResponse_RAADA_20230904.pdf')

model <- lm(MTX ~ Response, data = ada_mvals_aumc_svd_df)
summary(model)
```

No clear separation between responders and non-responders. Also no clear separation between responders and non-responders, when looking into linear regression also no significant result in the distribution of MTX usage in Responders/Non-responders

Differential methylation

Next up, we will look into differential methylation based on response.

```
gmset_anno <- minfi::getAnnotation(gmset_aumc_filtered)
gmset_anno_gr <- makeGRangesFromDataFrame(df = gmset_anno, keep.extra.columns = T,
    seqnames.field = "chr", start.field = "pos", end.field = "pos")

pData(gmset_aumc_filtered)$Response <- factor(pData(gmset_aumc_filtered)$Response,
    levels = c("Nonresponder", "Responder"))
design <- model.matrix(~Response + predictedSex + Age, data = pData(gmset_aumc_filtered))
colnames(design) <- c("(Intercept)", "Responder", "Male", "Age")

## linear model is fitted to the expression data for each probe (to describe
## relationship between variables)
mvals_lmfit <- lmFit(mvals_aumc, design = design)
betas_lmfit <- lmFit(betas_aumc, design = design)

## estimates probability distribution of data (M values and beta values)
mvals_ebayes <- eBayes(mvals_lmfit)
betas_ebayes <- eBayes(betas_lmfit)
topTable(betas_ebayes)
betas_treat <- treat(betas_lmfit)
topTreat(betas_treat)

## differentially methylated positions
dmps_rvnr <- topTable(mvals_ebayes, coef = "Responder", number = "Inf", adjust.method = "BH")

dmps_rvnr <- cbind(dmps_rvnr, Beta = coefficients(betas_ebayes)[rownames(dmps_rvnr),
    "Responder"], data.frame(gmset_anno_gr[rownames(dmps_rvnr), ]))
dmps_rvnr_sig <- dmps_rvnr[dmps_rvnr$adj.P.Val < 0.05, ]

dmps_rvnr_genes <- lapply(strsplit(dmps_rvnr$UCSC_RefGene_Name, ";"), unique)
names(dmps_rvnr_genes) <- rownames(dmps_rvnr)
dmps_rvnr_genes_nz <- dmps_rvnr_genes[which(lapply(dmps_rvnr_genes, length) != 0)]

dmps_rvnr_genes_nz_df <- data.frame(CpG = gsub("(^cg[0-9]{8}).+$", "\\1", names(unlist(dmps_rvnr_genes_nz))),
    Gene = unlist(dmps_rvnr_genes_nz))
dmps_rvnr_genes_nz_df$pvalue <- dmps_rvnr[dmps_rvnr_genes_nz_df$CpG, "P.Value"]

dmps_rvnr_genes_nz_list <- split(dmps_rvnr_genes_nz_df, dmps_rvnr_genes_nz_df$Gene)
dmps_rvnr_genes_nz_list <- dmps_rvnr_genes_nz_list[unlist(lapply(dmps_rvnr_genes_nz_list,
```

```
    nrow)) != 1]

dmps_rvnr_fisher <- lapply(X = dmps_rvnr_genes_nz_list, FUN = function(gene) {
    unlist(EmpiricalBrownsMethod::empiricalBrownsMethod(data_matrix = betas_aumc[which(rownames(gmset_aumc_filtered) %in%
        gene$CpG), ], p_values = gene$pvalue, extra_info = T))
})
rm(dmps_rvnr_genes_nz_list)

dmps_rvnr_ma <- data.frame(Gene = names(dmps_rvnr_fisher), do.call(rbind, dmps_rvnr_fisher))
colnames(dmps_rvnr_ma) <- c("Gene", "Brown_pval", "Fisher_pval", "Scale_Factor_C",
    "DF")
dmps_rvnr_ma$Brown_padj <- p.adjust(dmps_rvnr_ma$Brown_pval)
dmps_rvnr_ma <- dmps_rvnr_ma[order(dmps_rvnr_ma$Brown_pval), ]
dmps_rvnr_ma_sig <- dmps_rvnr_ma[which(dmps_rvnr_ma$Brown_padj < 0.05), ]
```

No significantly differentially methylated positions found based on response.

Predictor probes have been found in collaboration with HorAIzon, with the use of machine learning models built with gradient boosting and stability selection for prediction of response. In the following script, we will visualize the methylation levels of these predictor probes in heatmaps.

```r
hm_colanno <- data.frame(Response = pData(gmset_aumc_processed)$Response, row.names = colnames(gmset_aumc_processed))

heatmap <- pheatmap::pheatmap(betas_aumc[horaizon_markers$CGID, ], scale = "row",
    show_colnames = F, annotation_col = hm_colanno)
heatmap_27pred <- pheatmap::pheatmap(betas_aumc[secondary_split$CGID, ], scale = "row",
    show_colnames = F, annotation_col = hm_colanno)
heatmap_top15 <- pheatmap::pheatmap(betas_aumc[top15_xgb$CGID, ], scale = "row",
    show_colnames = F, annotation_col = hm_colanno)

save_pheatmap_pdf <- function(x, filename, width = 7, height = 7) {
    stopifnot(!missing(x))
    stopifnot(!missing(filename))
    pdf(filename, width = width, height = height)
    grid::grid.newpage()
    grid::grid.draw(x$gtable)
    dev.off()
}

save_pheatmap_pdf(heatmap, "heatmap_pred.pdf")
save_pheatmap_pdf(heatmap_27pred, "heatmap_27pred.pdf")
save_pheatmap_pdf(heatmap_top15, "heatmap_top15.pdf")
```

Visualisation of predictor probes’ methylation levels in boxplots:

```
Cairo(width = 2000, height = 1000, type = "pdf", file = "/mnt/smb/lpersonal/femke.mol/RA_ADA/output/sameorder_top15horaizonp
robes_boxplots_230828.pdf",
    bg = "white", units = "px", dpi = 120)

top15_horaizonprbs <- data.frame(betas_aumc[top15_xgb$CGID, ], ID = top15_xgb$CGID) %>%
    mutate(ID = fct_relevel(ID, "cg11553311", "cg16113156", "cg02408697", "cg13831575",
        "cg02613380", "cg11744538", "cg09419670", "cg09978860", "cg11939300", "cg21048050",
        "cg09817162", "cg27572370", "cg20561509", "cg20540428", "cg14178589")) %>%
    tidyr::pivot_longer(!ID, names_to = "SXSPOS", values_to = "Beta") %>%
    dplyr::mutate(SXSPOS = gsub("^X", "", SXSPOS)) %>%
    dplyr::left_join(data.frame(pData(gmset_aumc_filtered), SXSPOS = colnames(gmset_aumc_filtered)) %>%
        dplyr::select(SXSPOS, Response), by = "SXSPOS") %>%
    ggplot(aes(x = ID, y = Beta, col = Response)) + geom_boxplot(outlier.shape = NA,
    position = position_dodge(1)) + geom_point(position = position_jitterdodge(jitter.width = 0.1,
    dodge.width = 1), pch = 21, alpha = 0.2) + labs(title = "Adalimumab response in Rheumatoid arthritis",
    y = "%Methylation") + theme_bw() + scale_colour_brewer(palette = "Dark2") + theme(axis.text.x = element_text(angle = 90,
    vjust = 0.5, hjust = 1), axis.title.x = element_blank(), panel.grid.major = element_blank(),
    panel.grid.minor = element_blank(), legend.pos = "bottom")
```

(Gene) annotation of top CpGs (secondary split)

```
top27_gset <- gmset_aumc_processed[secondary_split$CGID, ]
cpg_top27 <- getAnnotation(top27_gset)
annotation <- as.data.frame(cpg_top27@listData)

args <- commandArgs(trailingOnly = TRUE)
if (length(args) != 2) {
    stop(paste0("Script needs 2 arguments. Current input is:", args))
}

# Install the Illumina HumanMethylation EPIC 10b5 (hg38) annotation
if (!"IlluminaHumanMethylationEPICanno.ilm10b5.hg38" %in% rownames(installed.packages())) {
    devtools::install_github("achilleasNP/IlluminaHumanMethylationEPICmanifest")
    devtools::install_github("achilleasNP/IlluminaHumanMethylationEPICanno.ilm10b5.hg38")
    require(IlluminaHumanMethylationEPICanno.ilm10b5.hg38)
} else {
    require(IlluminaHumanMethylationEPICanno.ilm10b5.hg38)
}

activepromoters_path <- args[1]
epic_annotation_csv_path <- args[2]

epic_anno <- minfi::getAnnotation(IlluminaHumanMethylationEPICanno.ilm10b5.hg38)
epic_anno$pos_hg38 <- as.numeric(epic_anno$Start_hg38) + 1
epic_anno <- data.frame(epic_anno) %>%
    dplyr::filter(!is.na(CHR_hg38))
epic_anno_hg38_gr <- makeGRangesFromDataFrame(epic_anno, keep.extra.columns = T,
    seqnames.field = "CHR_hg38", start.field = "pos_hg38", end.field = "pos_hg38")

epic_anno <- as.data.frame(epic_anno_hg38_gr) %>%
    dplyr::rename(chr_hg38 = seqnames, pos_hg38 = start) %>%
    dplyr::select(-end)
epic_anno_hg19_gr <- makeGRangesFromDataFrame(epic_anno, keep.extra.columns = T,
    seqnames.field = "chr", start.field = "pos", end.field = "pos")

pchic <- read.csv("/mnt/smb/lpersonal/femke.mol/RA_ADA/data/ActivePromoterEnhancerLinks.tsv",
    sep = "\t")
pchic_gr_bait <- makeGRangesFromDataFrame(pchic, keep.extra.columns = T, start.field = "baitSt",
    end.field = "baitEnd", seqnames.field = "baitChr")
```

```
pchic_gr_anno_bait <- annotatePeakInBatch(myPeakList = pchic_gr_bait, AnnotationData = genes(TxDb.Hsapiens.UCSC.hg19.knownGe
ne))
pchic_gr_anno_bait <- addGeneIDs(annotatedPeak = pchic_gr_anno_bait, orgAnn = "org.Hs.eg.db",
    feature_id_type = "entrez_id", IDs2Add = "symbol")
pchic_gr_anno_oe <- data.frame(pchic_gr_anno_bait) %>%
    dplyr::rename(baitChr = seqnames, baitSt = start, baitEnd = end) %>%
    makeGRangesFromDataFrame(keep.extra.columns = T, seqnames.field = "oeChr", start.field = "oeSt",
        end.field = "oeEnd")
epic_anno_hg19_gr$Enhancer_gene <- NA
pchic_epic_anno_hg19_gr_overlap <- findOverlaps(pchic_gr_anno_oe, epic_anno_hg19_gr)
epic_anno_hg19_gr$Enhancer_gene[subjectHits(pchic_epic_anno_hg19_gr_overlap)] <- pchic_gr_anno_oe[queryHits(pchic_epic_anno_
hg19_gr_overlap),
    ]$symbol

epic_anno <- data.frame(epic_anno_hg19_gr) %>%
    dplyr::rename(chr_hg19 = seqnames, pos_hg19 = start) %>%
    dplyr::select(-c(end, width))

annotated_cpgs <- epic_anno %>%
    filter(Name %in% secondary_split$CGID)
```

Hypothesis driven approach

We continue with the hypothesis driven approach, for which we look into genes known to play a role in RA: STAT3, CXCL12, IL-6, IL-10, DLGAP2 and PRSS16.

```
# Function made by A.Y.F. Li Yim
dmg_plot_continuous <- function(gene_of_interest, tophits_gr, pval_colname, xlim = NULL, ylim = NULL, smooth = T, stat = sta
t){
  require(ggbio)
  require(TxDb.Hsapiens.UCSC.hg19.knownGene)

  data(genesymbol, package = "biovizBase")

  cpgs <- tophits_gr[grep(paste0("(^|;)", as.character(gene_of_interest), "($|;)"), tophits_gr$UCSC_RefGene_Name),]

  ## methylation difference
  mdiff_track <- ggplot(cpgs, aes(x = start, y = Beta)) +
    geom_hline(yintercept = 0) +
    theme_bw() +
    theme(legend.pos = "top",
          axis.title.y = element_blank(),
          axis.text.y = element_text(hjust = -0.4),
          panel.grid.major = element_blank(),
          panel.grid.minor = element_blank(),
          text = element_text(size = 10),
          panel.border = element_blank())

  if(!is.null(xlim)) mdiff_track <- mdiff_track + xlim(xlim)
  if(!is.null(ylim)) mdiff_track <- mdiff_track + ylim(ylim)

  if(smooth){
    mdiff_track <- mdiff_track + geom_smooth(method = "loess")
  } else{
    mdiff_track <- mdiff_track + geom_line()
  }

  mdiff_track <- mdiff_track +
    geom_point(aes(size = -log10(eval(parse(text = pval_colname)))), alpha = 0.5) +
    scale_size_continuous(name = expression(paste(-log[10], "(p-value)")))

  gene_track <- ggplot() +
    geom_alignment(TxDb.Hsapiens.UCSC.hg19.knownGene, which = genesymbol[gene_of_interest] , stat = stat, label = FALSE, arr
ow.rate = 0.015) + #, gap.geom = "chevron") +
```

```
    theme_bw() +
    theme(panel.grid.major = element_blank(),
          panel.grid.minor = element_blank(),
          text = element_text(size = 12),
          panel.border = element_blank())

  if(!is.null(xlim)) gene_track <- gene_track + xlim(xlim)

  plotobj <- tracks("Gene" = gene_track,
                    "% Methylation difference" = mdiff_track,
                    heights = c(1, 4),
                    main = gene_of_interest)
  return(plotobj)
}

dmps_rvnr_gr <-  makeGRangesFromDataFrame(dmps_rvnr, keep.extra.columns = T, seqnames.field = "seqnames", start.field = "sta
rt", end.field = "end")

Cairo(width = 1200, height = 1400, type = "pdf", file = "/mnt/smb/lpersonal/femke.mol/RA_ADA/STAT3_reduced.pdf", bg = "whit
e", units = "px", dpi = 120)
dmg_plot_continuous(gene_of_interest = "STAT3",
                    tophits_gr = dmps_rvnr_gr,
                    smooth = T,
                    pval_colname = "P.Value",
                    stat = "reduce")
dev.off()

Cairo(width = 1200, height = 1400, type = "pdf", file = "/mnt/smb/lpersonal/femke.mol/RA_ADA/CXCL12_reduced.pdf", bg = "whit
e", units = "px", dpi = 120)
dmg_plot_continuous(gene_of_interest = "CXCL12",
                    tophits_gr = dmps_rvnr_gr,
                    smooth = T,
                    pval_colname = "P.Value",
                    stat = "reduce")
dev.off()

Cairo(width = 1200, height = 1400, type = "pdf", file = "/mnt/smb/lpersonal/femke.mol/RA_ADA/IL6_reduced.pdf", bg = "white",
units = "px", dpi = 120)
```

```
dmg_plot_continuous(gene_of_interest = "IL6",
                    tophits_gr = dmps_rvnr_gr,
                    smooth = T,
                    pval_colname = "P.Value",
                    stat = "reduce")
dev.off()

Cairo(width = 1200, height = 1400, type = "pdf", file = "/mnt/smb/lpersonal/femke.mol/RA_ADA/IL10_reduced.pdf", bg = "whit
e", units = "px", dpi = 120)
dmg_plot_continuous(gene_of_interest = "IL10",
                    tophits_gr = dmps_rvnr_gr,
                    smooth = T,
                    pval_colname = "P.Value",
                    stat = "reduce")
dev.off()

Cairo(width = 1200, height = 1400, type = "pdf", file = "/mnt/smb/lpersonal/femke.mol/RA_ADA/DLGAP2_reduced.pdf", bg = "whit
e", units = "px", dpi = 120)
dmg_plot_continuous(gene_of_interest = "DLGAP2",
                    tophits_gr = dmps_rvnr_gr,
                    smooth = T,
                    pval_colname = "P.Value",
                    stat = "reduce")
dev.off()

Cairo(width = 1200, height = 1400, type = "pdf", file = "/mnt/smb/lpersonal/femke.mol/RA_ADA/PRSS16_reduce.pdf", bg = "whit
e", units = "px", dpi = 120)
dmg_plot_continuous(gene_of_interest = "PRSS16",
                    tophits_gr = dmps_rvnr_gr,
                    smooth = T,
                    pval_colname = "P.Value",
                    stat = "reduce")
dev.off()
```

GSEA

Gene set enrichment was performed using the ChAMP package

```r
pheno <- gmset_aumc@colData@listData

champebGSEA <- champ.ebGSEA(beta = betas_aumc, pheno = pheno$Response, arraytype = "EPIC")
champebGSEA_P.df <- as.data.frame(champebGSEA$GSEA$`Rank(P)`)
champebGSEA_P_or.df <- champebGSEA_P.df[order(champebGSEA_P.df$adjP), ]
champebGSEA_P_or.df$pathway <- rownames(champebGSEA_P_or.df)

GSEA_pathways <- read_excel("GSEA_output.xlsx", col_names = FALSE)
colnames(GSEA_pathways) <- c("gsea", "Pathway", "Reference")
colnames(champebGSEA_P_or.df) <- c("nREP", "AUC", "P(WT)", "P(KPMT)", "adjP", "gsea")
champebGSEA_P_or.df$Pathways <- GSEA_pathways$Pathway
champebGSEA_P_or.df$Reference_pathways <- GSEA_pathways$Reference

## total set
ggplot(data = champebGSEA_P_or.df, aes(x = adjP, y = reorder(gsea, -adjP))) + geom_bar(stat = "identity") +
    theme_bw() + theme(axis.title.x = element_blank(), axis.text.x = element_blank(),
    axis.ticks.x = element_blank()) + scale_y_discrete(labels = pathnames)
ggsave("230712_gsea_barplot_NRvsR.png", width = 10, height = 14)

## top 20
top20 <- champebGSEA_P_or.df[1:21, ]
pathnames <- top20$Pathways

ggplot(data = top20, aes(x = adjP, y = reorder(gsea, -adjP), fill = adjP)) + geom_bar(stat = "identity") +
    theme_bw() + theme(axis.title.x = element_blank(), axis.text.x = element_blank(),
    axis.ticks.x = element_blank()) + scale_fill_gradient(name = "Adjusted P-value",
    labels = scientific_format()) + scale_y_discrete(labels = rev(pathnames)) + labs(y = "Pathways")
ggsave("230717_gseatop20_barplot_NRvsR_blue.pdf", width = 14, height = 14)
```

Pathways found with GSEA approach are mostly related to immune response pathways.
